# An Upper-Limb Power-Assist Exoskeleton Using Proportional Myoelectric Control

**DOI:** 10.3390/s140406677

**Published:** 2014-04-10

**Authors:** Zhichuan Tang, Kejun Zhang, Shouqian Sun, Zenggui Gao, Lekai Zhang, Zhongliang Yang

**Affiliations:** 1 College of Computer Science and Technology, Zhejiang University, Hangzhou 310027, China; E-Mails: ttzzcc@163.com (Z.T.); ssq@zju.edu.cn (S.S.); gzg@zju.edu.cn (Z.G.); zlkzhang@gmail.com (L.Z.); 2 College of Mechanical Engineering, Donghua University, Shanghai 201620, China; E-Mail: yzl@dhu.edu.cn

**Keywords:** upper limb, power-assist exoskeleton, proportional myoelectric control, pneumatic muscles, motion intention

## Abstract

We developed an upper-limb power-assist exoskeleton actuated by pneumatic muscles. The exoskeleton included two metal links: a nylon joint, four size-adjustable carbon fiber bracers, a potentiometer and two pneumatic muscles. The proportional myoelectric control method was proposed to control the exoskeleton according to the user's motion intention in real time. With the feature extraction procedure and the classification (back-propagation neural network), an electromyogram (EMG)-angle model was constructed to be used for pattern recognition. Six healthy subjects performed elbow flexion-extension movements under four experimental conditions: (1) holding a 1-kg load, wearing the exoskeleton, but with no actuation and for different periods (2-s, 4-s and 8-s periods); (2) holding a 1-kg load, without wearing the exoskeleton, for a fixed period; (3) holding a 1-kg load, wearing the exoskeleton, but with no actuation, for a fixed period; (4) holding a 1-kg load, wearing the exoskeleton under proportional myoelectric control, for a fixed period. The EMG signals of the biceps brachii, the brachioradialis, the triceps brachii and the anconeus and the angle of the elbow were collected. The control scheme's reliability and power-assist effectiveness were evaluated in the experiments. The results indicated that the exoskeleton could be controlled by the user's motion intention in real time and that it was useful for augmenting arm performance with neurological signal control, which could be applied to assist in elbow rehabilitation after neurological injury.

## 1. Introduction

In order to assist physically disabled or elderly people, to increase the strength of the upper limb and for self-rehabilitation purposes, various upper-limb power-assist exoskeletons and robots have been developed [1–9]. Kiguchi [1] proposed an electromyogram (EMG)-based impedance control method to control an upper-limb power-assist robot, which was simple and adaptable to any user. The results showed that the robot had an effective power-assist performance when users performed some aiming motions. Yagi [2] discussed an upper-limb power-assist system to assist workers with lifting a 30-kg rice bag without inducing lower back pain. The system used a pneumatic actuator to support shoulder and elbow movement. Su [3] presented electromyogram (EMG)-based neural network control of an upper-limb power-assist exoskeleton robot, which could predict the user's motion intention precisely. A four degrees-of-freedom system actuated by pneumatic muscles on the shoulder, elbow and wrist was built to assist the patients with achieving therapy at home or in the clinic [4], which was safe and easy to use. Rosen [5] constructed an exoskeleton structure, including two links and two joints, to demonstrate the feasibility of using an EMG-based control.

In most previous studies, surface electromyogram (EMG), as one of the neurological signals, is often used for the control signal of the power-assist exoskeletons, since it directly reflects the user's muscle activity level in real time [3,6–9]. However, most EMG control methods belong to on-off control (constant speed in one direction or full stop) [10–13]. The control method could not understand or estimate the user's motion intention in real time. However, real-time control is important, which could make the exoskeleton augment the power-assist performance effectively [14]. It is especially important for controlling an upper-limb power-assist exoskeleton, since the motion of the upper limb is complex in daily life. Proportional myoelectric control is different from on-off control, as it is a continuous and real-time control method to be used to control prostheses, orthosis and power-assist exoskeletons [15–18]. Ferris [19–21] built an ankle-foot orthosis actuated by two pneumatic muscles for studying human walking and assisting gait rehabilitation using proportional myoelectric control. Additionally, there were two other improved versions that offer torques to the knee and hip joints. Fougner [22,23] developed an upper limb prosthesis to evaluate the effects of limb position on pattern recognition by proportional myoelectric control. Some related tests of wrist rotation and hand opening/closing were done in this study. Muceli [24] proposed a proportional control strategy that could be practically applied in amputees for the real-time control of multiple degrees-of-freedom. Artificial neural networks were used as the control strategy to estimate the position of the complex wrist and hand movement. Pistohl [25] studied efficient ways of high-dimensional proportional myoelectric control according to many degrees-of-freedom hand prostheses and found some control principles in prosthetic applications.

In general, some of difficulties in controlling upper-limb power-assist exoskeleton based on EMG signals have been as follows: (1) traditional control methods do not predict users' motion intention, like the on-off control; (2) it is difficult to control an upper-limb exoskeleton in real time; (3) traditional control methods are not continuous, which makes user operation and adaption difficult; and (4) most of the traditional control methods belong to passive control. In order to solve these problems effectively, an upper-limb power-assist exoskeleton actuated by pneumatic muscles using proportional myoelectric control was constructed. An experiment protocol was established, including collecting the EMG signals and the elbow angle, which was used for recording data for the following experiments. With the feature extraction procedure and the classification (back-propagation neural network), an EMG-angle model was built to be used for pattern recognition. The angle that was predicted could be transferred to the control signals of the actuators (pneumatic muscles) according to the corresponding equations. The mapping from the input signal (EMG signal) to the output signal (voltage value) was set up. The upper-limb power-assist exoskeleton's control scheme and the power-assist effectiveness were evaluated and compared in four experiments.

This paper is organized as follows. In Section 2, we describe the hardware of the upper-limb power-assist exoskeleton and the experimental methods. Section 3 presents the results of the experiments and some data analyses. The reliability of the control scheme and the positive effectiveness of the power-assist were found in experimental data. Section 4 explains and discusses the experimental results. Section 5 draws our conclusions.

## 2. Methods

### 2.1. Subjects

Six male subjects (age = 25 ± 3 years, height = 171.0 ± 5.4 cm, weight = 62.1 ± 6.0 kg) participated in this study. All subjects had a medical examination to eliminate any musculoskeletal and nerve diseases. Before the experiment, all subjects were requested not to participate in any upper-limb activities that would lead to fatigue.

### 2.2. Hardware

**Figure 1.**
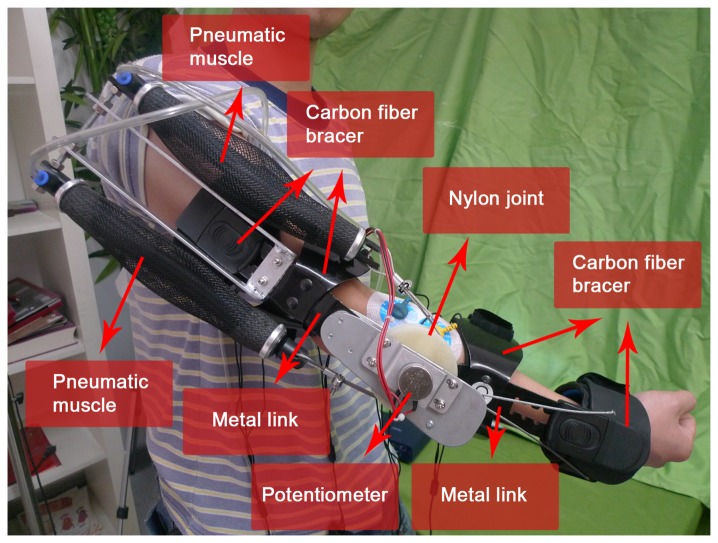
The overview of the upper-limb power-assist exoskeleton.

We fabricated an upper-limb power-assist exoskeleton for the subject's right arm, as shown in Figure 1. It consisted of two metal links corresponding to the arm limbs (the upper arm and the lower arm), a nylon joint corresponding to the elbow joint, four size-adjustable carbon fiber bracers, a potentiometer and two pneumatic muscles. The two links could adjust in the length for different subjects. The length of the upper and lower arm links were from 25 to 30 cm and from 20 to 25 cm, respectively. The joint was a 6-cm diameter nylon axis. As a one degree-of-freedom mechanism, the shoulder had a specific angle (*θ*_1_) from 0° to 180°, and the angle (*θ*_2_) range of the elbow joint could move from 0° to 145° (the average human anthropometric boundaries) [26]. The load of the exoskeleton was 2.1 kg.

The exoskeleton was fixed to the arm using four size-adjustable carbon fiber bracers (two for the upper arm and two for the lower arm). There were cotton linings clinging to the inside of the bracers, which allowed subjects feel comfortable in their movement. There was a potentiometer that consisted of a rotation axis, and a fixed end positioned the center of the nylon joint. The rotation axis connected to the nylon joint, and the fixed end connected to the upper arm link.

**Figure 2.**
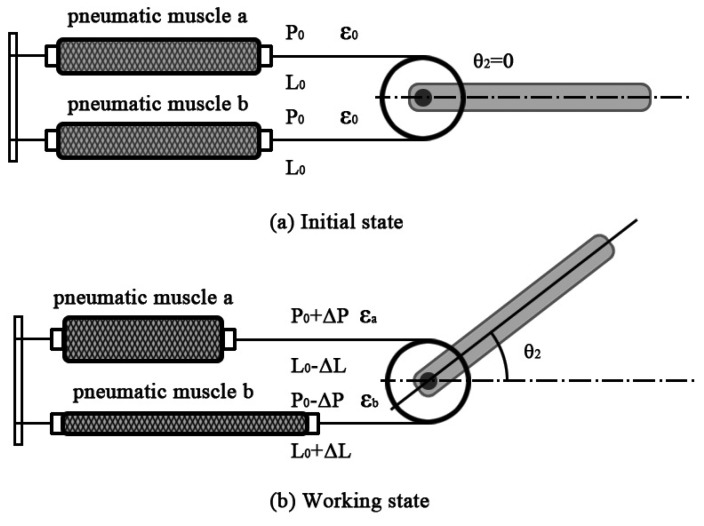
The elbow-joint model.

Two pneumatic muscles (SPCU-S-1, The Shadow Robot Company Ltd., London) connecting to the nylon axis could drive the rotation of the joint, as shown in Figure 2. The main parameters of the pneumatic muscle were as follows: the initial diameter was 20 mm; the maximum contraction rate was 25%; the initial braiding angle of the fiber was 25°; and the highest pressure was 0.6 MPa. The relationship among contraction force, air pressure and contraction rate was defined as [27]:

(1) $$F=P\left[a{\left(1-ɛ\right)}^{2}-b\right]$$

where $a=\frac{3\pi D_{0}^{2}}{4{\text{tan}}^{2}\theta_{0}}$, $b=\frac{\pi D_{0}^{2}}{4{\text{sin}}^{2}\theta_{0}}$, *D*_0_ was the initial diameter, *θ*_0_ was the initial braiding angle of fiber, *ε* was the contraction rate, *F* was the contraction force and *P* was the air pressure.

As shown in Figure 2a, in order to make the joint move in the maximum range, two pneumatic muscles' initial air pressure was set as *P*_0_, the contraction rate was ε_0_, the contraction force of both pneumatic muscles was F_0_ and the length was *L*_0_. When a pressure signal, Δ*P*, was input to the pneumatic muscles, the air pressure became *P*_0_ + Δ*P* and *P*_0_ − Δ*P*, the contraction rate became *ε_a_* and ε*_b_*, the contraction force became *F_a_* and *F_b_* and the length became *L*_0_ − Δ*L* and *L*_0_ + Δ*L* (Δ*L* was the changes of the length), as shown in Figure 2b. The joint would move because of the unbalance of the torques, and then, a new balancing of the torques occurred [28].

The changes of length Δ*L* and the contraction rates, *ε_a_*, ε*_b_*, were calculated by:

(2) $$\Delta L=\frac{\theta_{2}\pi R}{180{}^{\circ}}$$

where *R* is the radius of the nylon joint.

(3) $${ɛ}_{a}={ɛ}_{0}+\frac{\theta_{2}\pi R}{180{}^{\circ}L_{0}}$$

(4) $${ɛ}_{b}={ɛ}_{0}-\frac{\theta_{2}\pi R}{180{}^{\circ}L_{0}}$$

The contraction forces, *F_a_* and F*_b_*, were calculated by:

(5) $$F_{a}=\left(P_{0}+\Delta P\right)\left[a{\left(1-{ɛ}_{a}\right)}^{2}-b\right]$$

(6) $$F_{b}=\left(P_{0}-\Delta P\right)\left[a{\left(1-{ɛ}_{b}\right)}^{2}-b\right]$$

According to Equations (3)-(6), the pressure signal, Δ*P*, was calculated by:

(7) $$\Delta P=\frac{F_{a}}{2\left[a{\left(1-{ɛ}_{0}-\frac{\theta_{2}\pi R}{180{}^{\circ}L_{0}}\right)}^{2}-b\right]}-\frac{F_{b}}{2\left[a{\left(1-{ɛ}_{0}+\frac{\theta_{2}\pi R}{180{}^{\circ}L_{0}}\right)}^{2}-b\right]}$$

When the angle, *θ*_2_, was measured, the pressure signal, Δ*P*, could be calculated; as we can see from Equation (7), the relationship between them was nonlinear. There was a nonlinear relationship between the pressure signal and the control signal of the pneumatic muscles (voltage), and then, the control signal was gained according to the related conversion equations.

### 2.3. Experimental Protocol

Upon arrival, subjects were asked to have anthropometric measurements taken (age, height, weight), and then, the procedures and equipment used for these experiments were introduced to them. Before each experiment, the subject was requested to wear no shirt. There were two movements of the elbow joint: flexion and extension. The agonistic muscle and the synergistic muscle of the flexion movement were the biceps brachii and the brachioradialis [29]. The agonistic muscle and the synergistic muscle of the extension movement were the triceps brachii and the anconeus [29]. The four pairs of electrodes were attached to the four muscles of the right arm to collect the EMG signals in the experiments. The inter-electrode distance was 2 cm. The placement of electrodes was in the direction of the muscle fibers on the midline of the muscle belly and avoided the innervation zone of the muscles [30]. The position was marked with a pen to ensure the same positions on every experiment. Before electrode attachment, alcohol was used to clean the skin, and conductive gel was used to improve the contact of the electrode with the skin [31]. In each experiment, new electrodes were attached again on the pen mark.

After the surface electrodes were attached and the signal was normal, the upper-limb power-assist exoskeleton was attached to the subject's right arm. The angle of the shoulder (*θ*_1_) was 90°, and the angle of the elbow (*θ*_2_) was from 0° to 90°. There were a total of four experiments. In experiment one, the subject held a 1-kg load wearing the exoskeleton, but with no actuation, and performed the elbow flexion-extension movement under three different motion periods (a 2-s, 4-s and 8-s period). The EMG signals of four muscles and the angle of the elbow were collected. Every subject performed sixty sets of movement under every motion period. Three models (EMG-angle) corresponding to the three different motion periods were built. The reliability of the control strategy was evaluated. The conditions of the other three experiments were as follows: (1) in experiment two, the subject held a 1-kg load by hand without wearing the exoskeleton; (2) in experiment three, the subject held a 1-kg load wearing the exoskeleton, but with no actuation; (3) in experiment four, the subject held a 1-kg load wearing the exoskeleton under direct proportional myoelectric control. The model (EMG-angle) that had the best prediction performance in experiment one was used to predict the elbow angle and to control the exoskeleton. The contrast of the three experiments could evaluate the power-assist effectiveness.

### 2.4. Control Scheme

**Figure 3.**
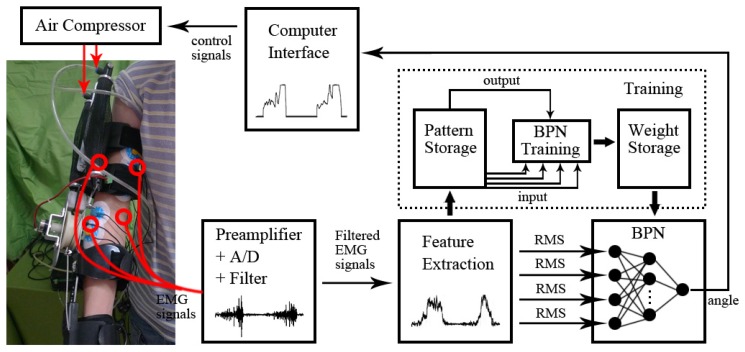
The control scheme based on the proportional myoelectric control. BPN, back-propagation neural network; EMG, electromyogram; RMS, root mean square.

As shown in Figure 3, a control scheme based on the proportional myoelectric control was constructed.

After the procedures of the preamplifier, the analog-to-digital converter and the filter, the raw EMG signal was transferred to the filtered EMG signal. However, it was not suitable as the input signals for the classifier (back-propagation neural network (BPN)); the feature of the signal should be extracted. As the time-domain indicator of EMG, the root mean square (RMS) was calculated and used as the feature. The RMS was calculated as [32]:

(8) $$\text{RMS}=\sqrt{\frac{1}{N}\sum_{i=1}^{N}v_{i}^{2}}$$

where *v_i_* is the voltage at the *i*-th sampling and *N* is the number of sampling points.

The back-propagation neural network (BPN) as the classifier was used for the pattern recognition. A three-layer BPN (input layer, hidden layer and output layer) was created using MATLAB. There were four nodes in the input layer corresponding to the four features of the EMG (RMS of four muscles), and there was one node in the output layer corresponding to the angle. The number of the hidden layer's nodes should not be too large or small, or it would affect the learning speed and the generalization ability of the network [33]. At the same time, the hidden layer's nodes had to be smaller than the input layer to prevent over train [34]. The nodes of the hidden layer were set to three. BPN had the ability to map the nonlinear properties through training. During training, some sets of data and classes were selected to adjust the weights in order to get the proper input-output relation. All sets of data were randomly selected to separate into the training (80%) sets and the testing (20%) sets. A sigmoid function was used for the transfer function when training the network. The output is calculated by:

(9) $$y=f\left(\sum w_{i}x_{i}\right)=\frac{1}{1+e^{-\left(\sum w_{i}x_{i}\right)}}$$

where *y* is the output, *x_i_* is the input, *w_i_* is the weighting factor attached to that input, *e* is the error function and f()is the transfer function.

When the training ended, an EMG-angle model was built, namely the mapping from the four muscles' EMG signals to the elbow angle was found. An elbow angle (output) could be predicted from the EMG signals (input) using the model in real time. Then, the value of angle was input to the computer interface. According to Equation (7), the angle could transfer to the air pressure of pneumatic muscles (the control signals of actuators). There was a nonlinear relationship between the air pressure and the control signal of pneumatic muscles (voltage), and then, the control signal was gained. The mapping from the input signals (EMG signal) to the output signal (voltage) was set up. A threshold was applied to eliminate the noise of the EMG signal. When the EMG signal was below the threshold, no signal (voltage = 0 V) was output. When the EMG signal was above the threshold, it indicated the start of the movement. A direct relation between the nervous system and the motion of the exoskeleton was founded. Subjects would adapt to the exoskeleton easily by using simple and smooth (proportional) control.

### 2.5. Data Collection

Four pairs of surface electrodes were used to collect the EMG data from the biceps brachii, the brachioradialis, the triceps brachii and the anconeus. The sensors (MyoScan sensor), which connected with electrodes, could record the EMG signals up to 1,600 micro-volts (µV) and an active range from 20 to 500 Hz. The EMG signals during four experiments were collected, amplified and transmitted by a ten-channel digital EMG system (FlexComp Infiniti System, Thought Technology Ltd., Canada). All EMG data were collected at 1,024 Hz. Then, the raw EMG signals were high-pass filtered (4th-order Butterworth; the cutoff frequency was 50 Hz), rectified and low-pass filtered (4th-order Butterworth; the cutoff frequency was 10 Hz). According to Equation (8), the filtered EMG signals were transferred to the RMS (time-domain signals of EMG). We applied the overlapped windowing technique to process data windowing [35]. We set the length of the window as 200 ms (feature extraction), and the processing time (classification) was often less than 50 ms. Therefore, the window length and the processing time should be less than 300 ms in total in order to allow that subjects do not feel any delay, which made the exoskeleton user friendly.

The potentiometer (RV30YN30S, TOCOS, Japan) positioned on the center of the nylon joint was used to measure the angle. The data were collected at 1,024 Hz. When the joint of exoskeleton moved in angle, the rotation axis of the potentiometer moved in the same angle, and the corresponding voltage was output at the same time [36]. According to an analog-to-digital converter, the voltage was transferred to the corresponding angle. The data of EMG and angle were collected and processed synchronously using MATLAB (MathWorks, Inc., Natick, MA, USA).

## 3. Results

### 3.1. The Evaluation of the Control Scheme

**Figure 4.**
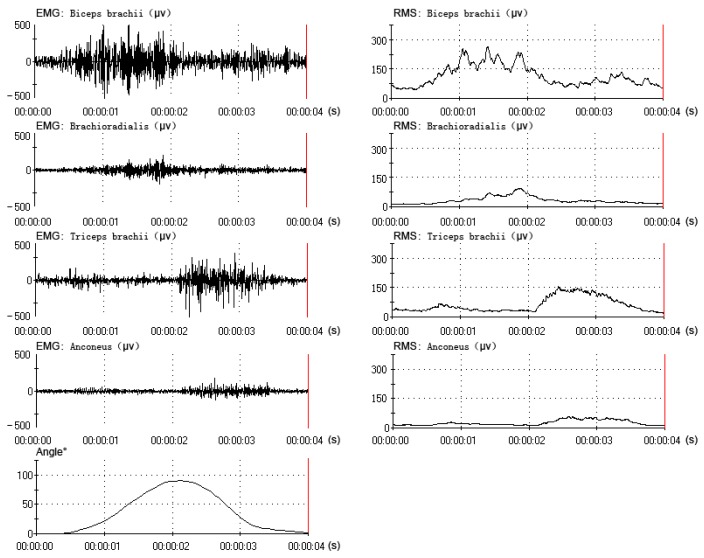
The muscles' EMG and RMS and the elbow angle under 2-s period (1–3 s).

**Figure 5.**
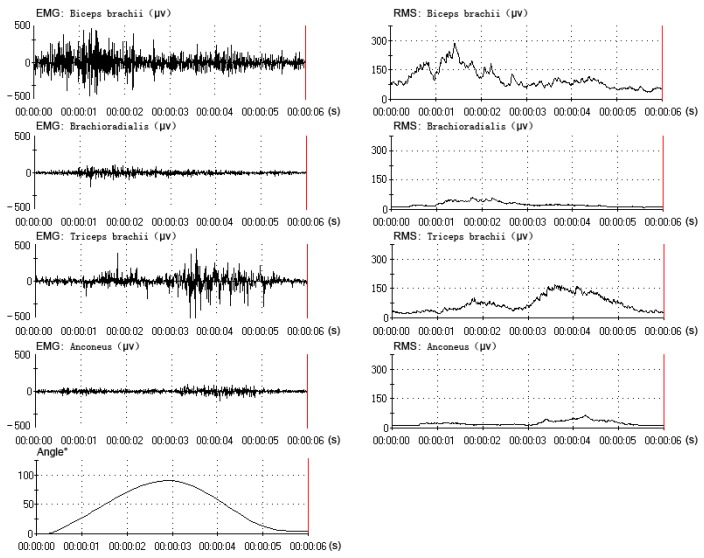
The muscles' EMG and RMS and the elbow angle under 4-s period (1–5 s).

**Figure 6.**
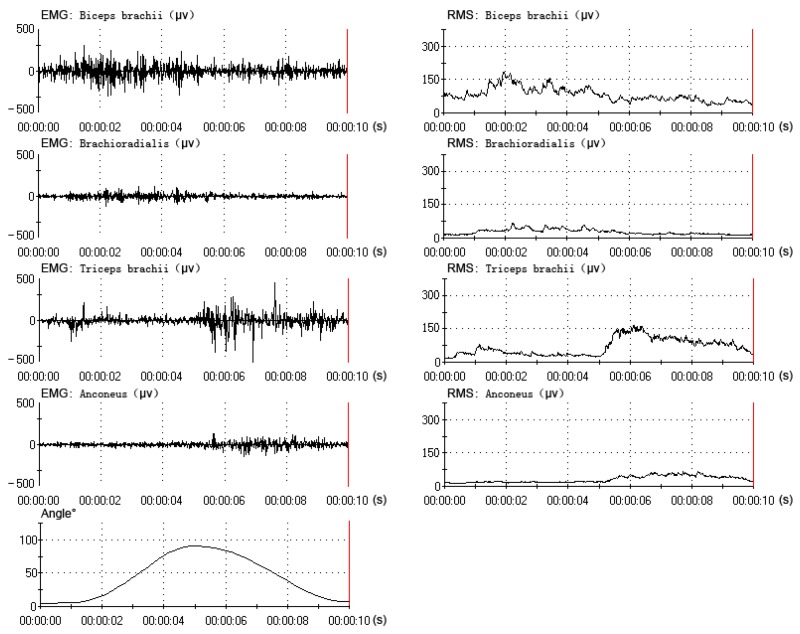
The muscles' electromyogram (EMG) and root mean square (RMS) and the elbow angle under the 8-s period (1–9 s).

All data were collected and processed from the same subject (one of the six subjects), who had better experimental performance (a better rhythm in the elbow flexion-extension movement). The data (EMG, RMS and angle) of one flexion-extension movement under three motion periods are shown in Figures 4, 5 and 6. The subject performed sixty flexion-extension movements under every motion period. Forty eight sets of data were randomly selected to form the training data, and the other twelve were selected to form the testing data. A total of three EMG-angle models (a 2-s, 4-s and 8-s period) were constructed.

The RMSE (root-mean-square error) and *R*^2^ were used to evaluate the prediction performance. The RMSE showed the error between the actual angle and the predicted angle, which could be calculated by:

(10) $$\text{RMS}E=\sqrt{\frac{1}{N}\sum_{n=1}^{N}{\left(A_{n}-A_{n}^{\prime }\right)}^{2}}$$

where *A_n_* is the predicted angle and $A_{n}^{\prime }$ is the actual angle. Normally, a smaller RMSE value (close to zero) and a larger *R*^2^ value (close to one) indicates that the prediction performance of the network is better. Linear regression was used to analyze the difference between the actual angle and the predicted angle (deviation rate). *k* and *j* were the slope and the intercept of the optimal regression line. The value of *k* was closer to one, and the value of *j* was closer to zero, meaning that there was a smaller error between the actual angle and the predicted angle.

The values of RMSE, *R*^2^, *k* and *j* for each network are shown in Table 1. For the different motion periods, the elbow movement under the four-second period had a lower RMSE (9.67) and greater *R*^2^ (0.87), when compared with the two-second period (RMSE: 10.70; *R*^2^: 0.83) and the eight-second period (RMSE: 12.42; *R*^2^: 0.79). Similar results were obtained from linear regression. The value of *k* (0.9107) was closer to one, and the value of *j* (4.4201) was closer to zero under the four-second period, when compared with the two-second period (*k*: 1.1985; *j*: −5.0340) and eight-second period (*k*: 0.8799; *j*: 10.6008). The prediction performance of the network under the four-second period was better than the other two, and the predicted angle was much more closely related to the actual angle.

**Figure 7.**
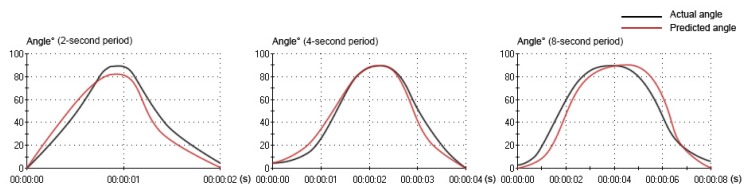
The curve of the actual angle and the curve of the predicted angle under three motion periods during one flexion-extension movement.

**Figure 8.**
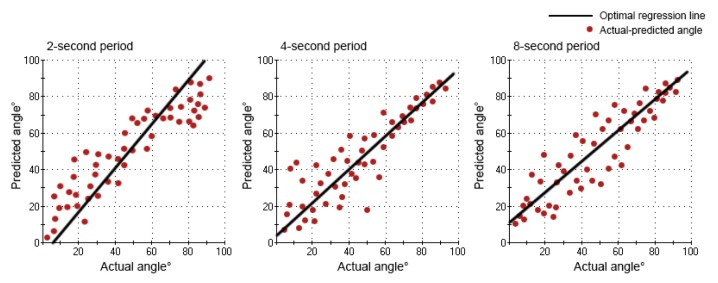
The optimal regression line of the actual angle and the predicted angle under three motion periods during flexion movement.

The relation between the actual angle and the predicted angle can be seen in Figures 7 and 8. In the movement under the four-second period, the curve of the predicted angle was capable of reflecting the curve of the actual angle well. The error between the actual angle and the predicted angle was smaller, but there was a little offset. In the movement under the two-second period, there was a larger error between the actual angle and the predicted angle. From 0° to 70°, the predicted angle was larger than the actual angle. From 70° to 90° and 90° to 0°, the predicted angle was lower than the actual angle. Especially from 80° to 90°, there was the largest error. In the movement under the eight-second period, there was a larger error between the actual angle and the predicted angle, as well. From 0° to 90°, the predicted angle was lower than the actual angle. From 90° to 0°, the predicted angle was larger than the actual angle. Especially from 90° to 40°, there was the largest error. However, the curve of the predicted angle could roughly reflect the direction and trend of the curve of the actual angle in all movements (2-s, 4-s and 8-s period).

### 3.2. The Evaluation of Power-Assist Effectiveness

**Figure 9.**
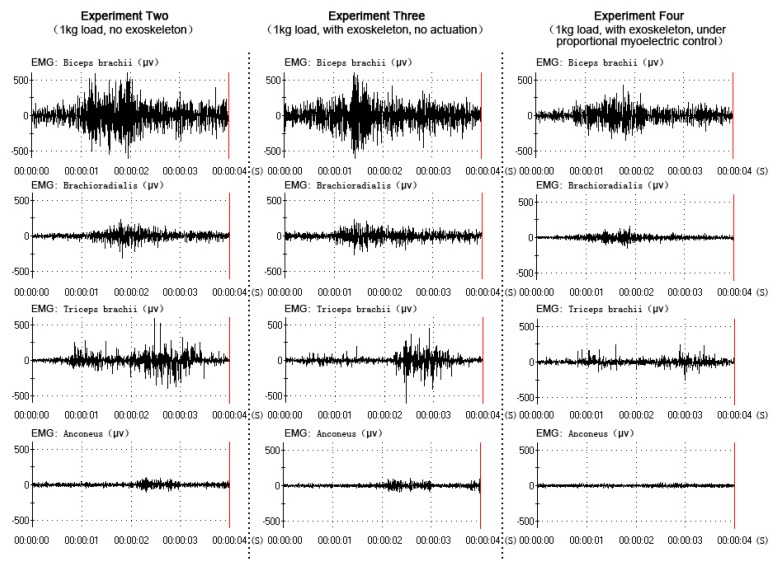
The four muscles' EMGs of the three experiments.

**Figure 10.**
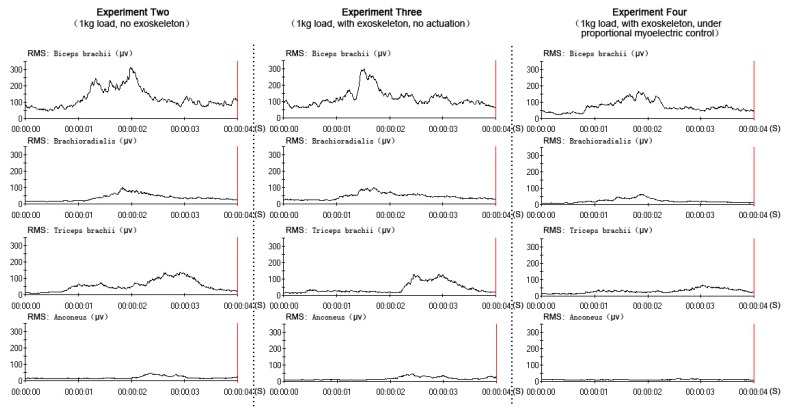
The four muscles' RMSs of the three experiments.

All data were collected and processed from the same subject (one of the six subjects), who had better experimental performance (a better rhythm in the elbow flexion-extension movement). The EMG and RMS output in three experiments (experiment two, experiment three and experiment four) were compared to verify the power-assist effectiveness, as shown in Figures 9 and 10. In the three experiments, the subject performed the elbow flexion-extension movement under the four-second period (this was the best prediction performance of the network). The amplitude of the four muscles' EMG and RMS was similar in experiment two and experiment three, which meant that the muscle activity was similar when the subject held a 1-kg load without the exoskeleton and with the exoskeleton, but with no actuation. The amplitude of the four muscles' EMG and RMS were decreased while wearing the exoskeleton under direct proportional myoelectric control (experiment four), which verified the positive power-assist effectiveness.

## 4. Discussion

This study demonstrated that there was the best prediction performance of the network (EMG-angle) under the four-second period. When one flexion-extension motion period was short (two-second period), the motion acceleration would affect the EMG amplitude. The higher speed would lead to the use of greater force, so the larger EMG amplitude was tested. Because muscle force reached the maximum value and the minimum value when the acceleration reached the maximum value and the minimum value, the functional state and the motion state of the nervous system would be in an unstable region, which led to a worse prediction performance. When one flexion-extension motion period was long (eight-second period), the speed, which was not constant during the motion period, would affect the EMG amplitude. Because the speed could not be constant in a long motion period, the muscle force of subjects could not maintain an isometric level, which led to a worse prediction performance. as well. Keeping constant motion speed and rhythm would obtain the good prediction performance. Several previous studies had discussed the relation between acceleration, speed and prediction performance. Fougner [23] thought that the nervous system could control the angles and positions of the many parts of the arm, and it could control the speed and muscle force, as well. However, the nervous system could not keep the constant motion speed and the isometric muscle force during a short or long period without external equipment. Au [37] used EMG signals to evaluate the performance of a time-delayed artificial neural network (TDANN) to predict the shoulder and elbow motions. There was a 20° difference between the maximum RMSE and the minimum RMSE at the different speeds and different accelerations. Lee [38] studied human intention during a lifting movement (up/down movement) from EMG signals with different speeds (0.2, 0.3, 0.4 and 0.5 Hz). The movement direction was estimated with higher accuracy at speeds of 0.3 Hz (93.6%) and 0.4 Hz (94.8%), but with lower accuracy at speeds of 0.2 Hz (80.1%) and 0.5 Hz (83.0%). Motion acceleration and speed would affect the prediction performance to some extent. The relations between the acceleration and speed and the prediction performance will be discussed in our future studies.

The results in Figures 9 and 10 demonstrated a clear reduction in four muscles' EMG amplitude while wearing the exoskeleton under direct proportional myoelectric control (experiment four). However, there were many factors affecting the relation between the EMG amplitude and the muscle force [39], like muscle length, muscle contraction velocity and fatigue [40]. One subject was requested to perform elbow flexion-extension movement continuously (more than two hundred times in experiment one) and hold a 1-kg load in experiments two, three and four, which might have led to the muscle fatigue. Muscle fatigue could induce changes in the EMG signals: the amplitude and RMS (root mean square) of EMG would be increased [41]; the MF (median frequency) and MPF (mean power frequency) of EMG would be decreased [42]. These changes could affect the prediction performance of the network and the power-assist effectiveness. Muscle fatigue should be considered an important factor in our future studies.

The actuator of the upper-limb power-assist exoskeleton in this study was the artificial pneumatic muscle. Due to the greater range of motion at the elbow (from 0° to 145°), the contraction rate of the pneumatic muscle needed to be considered. However, pneumatic muscle could only shorten or lengthen about one third of the initial length. It could not be stretched beyond its limit length, which led to a limit on the range of motion. We reduced the diameter of the nylon joint and made the range of motion be from 0° to 90°, which was still less than the average human anthropometric boundaries. The portability and flexibility of the exoskeleton was another limitation. Due to the heavy air supply equipment (air pump), the subject could not move around wearing the exoskeleton. Future designs could choose different actuators (servo motors or hydraulic cylinders) to alleviate these limitations.

According to the above results of our study and previous studies, proportional myoelectric control had some advantages and disadvantages for the control of an upper-limb power-assist exoskeleton compared to the other control methods. The advantages were: (1) it provided an effective way to control the exoskeleton in real time using biological signals [43]; (2) it led to a greater mechanical assistance to improve the power-assist effectiveness; and (3) the nervous system could easily adapt the exoskeleton control for different motions [40]. The disadvantages were: (1) it was hard to obtain the same EMG signals from one motion, even with one person [1]; and (2) every subject needed to go through the system training (BPN) to recognize the input signals, due to the individual differences of subjects. The development of EMG electrodes and the optimization of control algorithms could overcome these disadvantages in the near future. At the same time, according to the state-of-the-art in this field, using EMG signals to control an upper-limb exoskeleton faces challenges for clinical use. Scheme and Englehart [44] thought that the dexterity with which one might control an exoskeleton had progressed very little, especially when controlling multiple degrees of freedom. Using pattern recognition to discriminate multiple degrees of freedom had shown great promise, but it had yet to transition to a clinically viable option. Jiang [45] found that myoelectric control had a great potential for improving the quality of life of persons with limb deficiency; however, its clinical and commercial impact is still limited. Given the difficulty of robust control solely by using EMG, the use of other sensor modalities seems necessary for the control of complex devices. The sensor-fusion approach would be the research direction in our future studies.

## 5. Conclusions

In this paper, we developed an upper-limb power-assist exoskeleton actuated by pneumatic muscles. The proportional myoelectric control method was proposed to control the exoskeleton according to the user's motion intention in real time. The elbow angle was estimated based on the EMG signals in the proposed method. A back-propagation neural network (BPN) was applied to construct the EMG-angle model to make the exoskeleton adaptable to every subject. There were a total of four experiments in this study. In experiment one, the reliability of the control scheme was evaluated according to the prediction performance of networks under different motion periods, and there was the best prediction performance under the four-second period. In experiments two, three and four, the power-assist effectiveness was evaluated under different conditions, and a positive effect was verified. The results indicated that the exoskeleton could be controlled by the user's motion intention in real time and that it was useful for augmenting arm performance with neurological signal control, which could be applied to assist with elbow rehabilitation after neurological injury.

## Figures and Tables

**Table 1.** The performance of three networks (2-s, 4-s and 8-s period).

	**2-s Period**	**4-s Period**	**8-s Period**
*RMSE*	10.70	9.67	12.42
*R*^2^	0.83	0.87	0.79
*k*	1.1985	0.9107	0.8799
*j*(°)	−5.0340	4.4201	10.6008
